# Vitamin D Supplementation Effects on Markers Related with Endothelial Function and Coagulation in Obese Orthopedic Patients: Insights from Acute and Chronic Cases

**DOI:** 10.3390/nu17050882

**Published:** 2025-02-28

**Authors:** Michał Gawryjołek, Michał Wiciński, Marta Michalska Gawryjołek, Jan Zabrzyński

**Affiliations:** 1 Department of Orthopaedics and Traumatology, Dr L. Blazek Multi-Specialty Hospital, 88-100 Inowroclaw, Poland; 2 Department of Pharmacology and Therapeutics, Faculty of Medicine, Collegium Medicum in Bydgoszcz, Nicolaus Copernicus University, M. Curie 9, 85-090 Bydgoszcz, Poland; michal.wicinski@cm.umk.pl; 3 Department of Radiology, Dr L. Blazek Multi-Specialty Hospital, 88-100 Inowroclaw, Poland; michalskamarta86@gmail.com; 4 Department of Orthopaedics and Traumatology, Faculty of Medicine, Collegium Medicum in Bydgoszcz, Nicolaus Copernicus University in Toruń, 85-092 Bydgoszcz, Poland

**Keywords:** orthopedic conditions, coagulation, endothelial function, vitamin D, obesity

## Abstract

Obesity is a risk factor for thrombosis-related diseases and a condition that leads to vitamin D deficiency. Furthermore, orthopedic conditions are also at risk for diseases associated with coagulation and endothelial function. This study aimed to assess whether vitamin D supplementation in patients with acute (AOCs) and chronic orthopedic conditions (COCs) and coexisting obesity could affect coagulation and endothelial function. Thirty-three obese individuals with AOCs or COCs were included in the study. Patients were supplemented with vitamin D at 4000 IU/day for 3 months. An enzyme-linked immunosorbent assay (ELISA) was used to measure the concentrations of alpha 2-antiplasmin (α2AP), vascular cell adhesion molecule 1 (VCAM-1), plasminogen activator inhibitor-1 (PAI-1), tissue factor pathway inhibitor (TFPI), and vitamin D, which were examined at two time points—before and after supplementation. Regardless of the increase in serum vitamin D levels in both groups after supplementation, there was a statistically significant increase in VCAM-1 and PAI-1 levels in the group with AOCs, whereas only VCAM-1 increased statistically significantly in the second group. For obese patients with COCs, vitamin D does not appear to have a potentially beneficial effect on coagulation and the endothelium.

## 1. Introduction

Obesity and overweight are conditions resulting in more significant health risks, in which excessive or abnormal body fat are crucial components [1]. Lin et al. mentioned that obesity increases the likelihood of various diseases, such as, for example, cardiovascular diseases (CVDs), metabolic syndrome, type 2 diabetes, osteoarthrosis, cancer, and depression [2]. In addition, one consequence of obesity is an increased incidence of deep venous thrombosis (DVT) and pulmonary embolism (PE) [3]. Ageno et al. in their meta-analysis showed that the risk of developing venous thromboembolism (VTE) is up to 2.33-fold higher in obese individuals [4]. Moreover, increased adipose tissue mass, related to obesity, is associated with higher levels of prothrombotic molecules, such as factor VII, fibrinogen, and tissue factor, due to systemic inflammation [5]. Similarly, plasminogen activator inhibitor-1 (PAI-1) is elevated in obesity, possibly related to increased PAI-1 expression in visceral tissue [6].

The risk of vitamin D deficiency is markedly increased in obese individuals, and studies show that vitamin D levels are inversely associated with obesity incidence. However, its effect on visceral fat loss is not fully understood [7].

Vitamin D deficiency is described as its serum level being under 20 ng/mL. Furthermore, the adequate level should be 40–60 ng/mL [8]. Studies have shown that an active form of vitamin D acts as a ligand through vitamin D nuclear receptors (VDRs) [9,10,11]. Apart from regulating calcium and phosphate levels in bones, these receptors play essential roles in tissues such as skeletal muscles and vascular endothelial cells [12,13,14]. These receptors mainly regulate diseases such as intestinal, renal, bone, skin, and cardiac disease [15]. The wide range of activities in different tissues mediated by VDRs results in an elevated risk of metabolic diseases and CVDs [9]. Moreover, vitamin D is believed to be involved in adipose tissue metabolism, particularly in adipogenesis, adipocyte diversification, energy homeostasis, and inflammatory processes. Importantly, due to its anti-inflammatory and antioxidant properties, vitamin D can prevent vascular endothelial damage [16,17,18]. It has also been shown that the vitamin can inhibit the coagulation pathway and, therefore, has an anticoagulant effect [19,20]. Hence, it appears that vitamin D deficiency may potentially impact the occurrence of a prothrombotic state, which is associated with the development of VTE [16].

Osteoarthritis (OA) is a joint disease that is a significant cause of pain and disability [21]. The findings from the Wuchuan Osteoarthritis Study indicate that nearly all (96.8%) of the mortality associated with knee OA can be attributed to its impact on mobility disability [22]. Meanwhile, results from the Osteoarthritis Initiative revealed that 22.4% of the effect of symptomatic knee OA on overall mortality was mediated by physical disability. In comparison, impairments influenced 26.5% of the physical component summary scores related to quality of life [23,24,25]. Other studies have also shown that hand OA may be a factor in the increased risk of cardiovascular events, which appears to be related to the systemic inflammation that is associated with OA [26]. Zeng et al. demonstrated in their study that while knee or hip OA is linked to a higher risk of VTE, hand OA does not show this association [27]. Despite several studies analyzing the association between OA and the risk of cardiovascular events, the issue seems to remain unresolved due to inconsistent results [25,28,29,30,31,32,33,34,35,36]. Moreover, venous return is slowed in patients with acute orthopedic diseases like bone fractures with additional limb immobilization, making them susceptible to spontaneous intravascular clotting [37].

Considering vitamin D’s pleiotropic properties and therapeutic function, it was decided to test whether it could affect coagulation and vascular endothelium markers in orthopedic patients with coexisting obesity. The objective was to determine whether high-dose vitamin D supplementation might influence these markers differently based on the type of orthopedic condition—acute or chronic.

## 2. Materials and Methods

### 2.1. Bioethics

The research adhered to the policies established by *Basic & Clinical Pharmacology & Toxicology* concerning both experimental and clinical studies. The investigation was conducted following the Declaration of Helsinki regarding experiments involving human subjects, following the approval granted by the Bioethics Committee of the Collegium Medicum in Bydgoszcz, Nicolaus Copernicus University in Toruń (approval number: KB 465/2022, approval date: 27 September 2022). All participants provided informed consent to participate in the study and were volunteers.

### 2.2. Study Cohort

Thirty-three patients from the Department of Orthopedic Surgery participated in the study. The selection of subjects was based on specific inclusion criteria: participants were required to be aged between 18 and 75 years, have a BMI greater than 30 kg/m^2^, and exhibit vitamin D deficiency levels below 30 ng/mL. Exclusion criteria encompassed pregnancy, regular anticoagulant use, cancer, dialysis, and liver disease. Additionally, none of the patients had previously taken vitamin D supplements.

The study participants were categorized into two groups: acute orthopedic conditions (AOCs) and chronic orthopedic conditions (COCs). The AOCs group consisted of patients suffering from bone fractures, joint sprains, and meniscal damage in the knee joint. The COCs group included individuals diagnosed with OA of the knee and hip. None of the patients were hospitalized. Patients with AOCs initially received emergency care before further treatment was implemented approximately one week after the fracture at the orthopedic clinic. Fractures include the proximal end of the humerus and the distal end of the radius.

To eliminate the effect of UV-B radiation on the results obtained, the study was conducted from September 2022 to May 2023. In addition, all participants maintained their usual eating habits throughout the study. Data on each subject’s age, weight, and height were also analyzed.

### 2.3. Samples Collection

Patients ingested vitamin D orally at 4000 IU daily for three months. Vitamin D concentration in serum was assessed in the hospital laboratory associated with the orthopedic clinic.

Each participant’s peripheral venous blood specimen was collected at two different time intervals—before and after the completion of the three-month vitamin D supplementation. Blood samples were taken from patients at a specified time in the morning while they were fasting and seated. The separated serum from these samples was promptly transported for further examination to the Department of Pharmacology and Therapeutics, Collegium Medicum in Bydgoszcz, Poland.

### 2.4. Outcome Evaluation

Serum and plasma samples were promptly prepared following standard protocols. The samples were frozen at −20 °C and transported on dry ice to the Department of Pharmacology and Therapeutics, where they were stored at −70 °C until analysis. The study determined markers—α2AP, TFPI, PAI-1, and VCAM-1—which play a role in thrombosis and homeostasis, as well as providing information on inflammation in the body. α2AP is an essential inhibitor of fibrinolysis. TFPI is a key regulator of the extrinsic coagulation pathway, where it inhibits coagulation by forming complexes with factor Xa and tissue factor, thereby preventing clot formation. PAI-1 regulates fibrinolysis, and its levels increase in conditions with an increased risk of thrombosis. VCAM-1, a marker of endothelial dysfunction, provides valuable information on inflammation and cardiovascular disease. Serum protein markers were assessed in all patients using an enzyme-linked immunosorbent assay (ELISA), with ELISA microplates provided by Sunredbio (SRB) Technology, Shanghai, China. Following the manufacturer’s guidelines, analyses were conducted using an EPOCH microplate spectrophotometer from BioTech, Santa Clara, CA, USA.

### 2.5. Statistical Analysis

Statistica 13.3 software was utilized to conduct the statistical analysis. The results for each group are presented as mean ± standard error of the mean (SEM). The normality of the distribution was assessed using the Shapiro–Wilk test. For each group, comparisons of markers and vitamin D concentrations before and after supplementation were made using either the Wilcoxon test or the *t*-test for dependent samples. The Mann–Whitney U-test or *t*-test for independent samples was employed to compare independent groups, specifically AOCs vs. COCs. Additionally, within the group experiencing AOCs, markers and vitamin D concentrations were analyzed by sex using these same tests. Spearman’s rank correlation was applied to evaluate the association between marker concentrations, age, BMI, and the subjects’ sex. Results with a *p*-value of less than 0.05 were deemed statistically significant.

## 3. Results

The study involved 33 obese patients, categorized based on their orthopedic conditions into two groups: those with AOCs (18 subjects) and those with COCs (15 subjects). In the AOCs group, the sex distribution was 34% male and 66% female. However, in the COCs group, 93% of subjects were females. Statistical analysis revealed that patients with COCs were significantly older than those in the AOCs group, with average ages of 63.47 ± 2.29 years compared to 53.22 ± 2.4 years (*p* = 0.002). In contrast, regarding mean BMI, the two groups were not statistically significantly different (33.61 ± 0.53 kg/m^2^ for the AOCs group and 33.67 ± 0.61 kg/m^2^ for the COCs group). No statistical differences in BMI were observed before and after the 3-month supplementation in any of the study groups.

Spearman’s rank correlation was conducted to explore potential relationships between markers, vitamin D levels, BMI, and patient age within each group (Table 1 and Table 2). For the AOCs group, possible correlations by patient sex were also analyzed.

The correlation analysis indicated no significant relationships between BMI, sex, the selected markers, and vitamin D in the AOCs group. In contrast, it showed that there was a negative statistically significant correlation between age and PAI-1 (R = −0.74; *p* < 0.05) and VCAM-1 (R = −0.53; *p* < 0.05) concentrations in the group with AOCs. The COCs group, on the other hand, showed no significant correlations of markers and vitamin D with the parameters studied (BMI, age).

In the AOCs group (Table 1), after three months of vitamin D supplementation there was a statistically significant increase in PAI-1 (2.51 ± 0.32 ng/mL vs. 2.82 ± 0.38 ng/mL; *p* = 0.043), VCAM-1 (13.22 ± 0.52 ng/mL vs. 14.13 ± 0.6 ng/mL; *p* = 0.007), and vitamin D (19.44 ± 1.4 ng/mL vs. 33.83 ± 3.12 ng/mL; *p* < 0.001) levels.

After 3 months of vitamin D supplementation in the group with COCs (Table 2), a statistically significant increase was observed in VCAM-1 levels (from 12.52 ± 0.38 ng/mL to 13.57 ± 0.61 ng/mL; *p* = 0.031) as well as in vitamin D concentrations (from 20.39 ± 1.66 ng/mL to 35.22 ± 2.46 ng/mL; *p* < 0.001).

Table 3 and Table 4 compare α2AP, TFPI, PAI-1, VCAM-1, and vitamin D levels in the AOCs and COCs groups before and after supplementation.

The comparison of markers and vitamin D concentrations revealed no statistically significant differences between the AOCs and COCs groups, regardless of whether it was before or after supplementation.

Additional statistical analyses were performed to see if the sex could have significantly affected serum markers. In the AOCs group, the ratio of males to females was 1:2. Regarding BMI values, the subjects did not differ statistically significantly. However, males were statistically markedly older than females in this group (61.0 ± 1.63 years vs. 49.33 ± 2.93 years; *p* = 0.017).

Differences between markers and vitamin D concentrations by sex in the AOCs group are shown in Table 5 (before supplementation) and Table 6 (after supplementation).

Before supplementation, no statistically significant differences in the concentrations of the parameters between males and females were observed in the AOCs group.

After completing supplementation, females with AOCs had higher PAI-1 levels than males in this group (3.37 ± 0.5 ng/mL vs. 1.7 ± 0.13 ng/mL; *p* = 0.03). The other showed no statistically significant difference.

Due to the significant sex disparity, the concentrations of markers and vitamin D in females in both groups before (Table 7) and after (Table 8) supplementation were also compared. Females in the COCs group were statistically significantly older than those in the AOCs group (63.14 ± 2.43 years vs. 49.33 ± 2.93 years; *p* = 0.002).

The above results show that there are no statistically significant differences in markers and vitamin D concentrations between females in the AOCs and COCs groups, both before and after supplementation.

## 4. Discussion

Our study aimed to test whether vitamin D could affect coagulation and the vascular endothelium in obese patients with orthopedic conditions. This was evaluated by changes in markers such as VCAM-1, PAI-1, α2AP, and TFPI. Participants in the study were categorized into two groups according to their orthopedic diseases—acute or chronic. The subjects received a daily dose of 4000 IU of vitamin D for three months and were double-checked for serum vitamin D levels before and after completing supplementation.

In conclusion, both groups had a statistically significant increase in serum vitamin D levels after 3 months of supplementation compared to baseline, where all patients were in the deficiency group. A statistically significant increase in VCAM-1 and PAI-1 was observed after vitamin D supplementation in the AOCs group, while only VCAM-1 increased statistically significantly in the COCs group. In contrast, no changes were observed in the concentrations of the other markers in both groups after supplementation. Moreover, comparing patients from the AOCs group with those from the COCs group showed no statistical differences in the concentrations of the studied markers before and after supplementation.

In addition, due to the large discrepancy in the sex distribution, especially in the group with AOCs, several additional statistical analyses were performed. Females in this group were statistically significantly younger than males. After supplementation, females in the AOCs group had statistically significantly higher PAI-1 levels than males. In contrast, comparing females in the AOCs and COCs groups revealed no significant differences in markers and vitamin D levels before and after supplementation. However, females in the COCs group were significantly older.

Patients with COCs were significantly older than those with AOCs. Interestingly, markers like VCAM-1 and PAI-1 in the latter group exhibited a statistically significant negative correlation with age. This may be due to the acute inflammatory response triggered, for example, by a fracture in AOCs patients. In contrast, COCs patients experience a chronic inflammatory reaction in their bodies. Of course, the influence of genetic and endocrine-related factors in both patient groups cannot be overlooked.

Obesity is one of the factors that induce endothelial dysfunction [38]. Saturated fatty acids also trigger an acute inflammatory response, enhancing the expression of intercellular adhesion molecule 1 (ICAM-1) and VCAM-1 [39]. A range of studies indicates that obesity, especially when coupled with other metabolic disorders known as metabolic or insulin resistance syndrome, significantly raises the risk of atherosclerosis and related complications [40,41]. Growing evidence points to persistent subclinical inflammation being a key factor in the development of obesity and its complications, including insulin resistance, dyslipidemia, and atherosclerosis [42]. Interestingly, there is a widely noted inverse relationship between vitamin D deficiency and obesity recognized. Many studies have confirmed the link between vitamin D deficiency and obesity, along with obesity-related diseases. However, the causal relationship remains uncertain. Several explanations exist for the inverse relationship with increased adiposity, yet none fully clarify this connection [43].

Bošanská et al. demonstrated that obesity correlates with elevated mRNA expression and protein levels of adhesion molecules such as ICAM-1 and VCAM-1 [44]. Numerous studies indicate a link between obesity and hemostatic alterations. Obese individuals displayed heightened levels of PAI-1 [45,46]. Recently, it has been noted that even adipose tissue may play a role in the increased PAI-1 levels in insulin resistance. Furthermore, pro-inflammatory cytokines are implicated in the augmented release of anti-fibrinolytic agents, such as PAI-1 and tissue factors, from the liver and adipose tissue [47,48]. PAI-1, a key regulator of fibrinolysis, is found in various tissues and cell types, including macrophages/monocytes, hepatocytes, the vascular endothelium, the adipose tissue in the heart and lungs, and platelets [49,50]. Recently, Cura-Esquivel et al. revealed that overweight and obese children had higher PAI-1 levels compared to their normal-weight counterparts [51]. Based on the above studies, obesity may be a predisposing factor for higher levels of the markers studied in our work.

Tabrizi et al., in their systematic review, analyzed fourteen clinical trials with 1253 patients included. No considerable effect of vitamin D supplementation was found on ICAM-1 and VCAM-1 levels [52]. Conversely, Naeini et al. demonstrated that vitamin D supplementation in a placebo-controlled, double-blind clinical trial led to a decrease in ICAM-1 and VCAM-1 among hemodialysis patients. However, there were no meaningful changes in the relationships between dosages, types of vitamin D administered, participants’ BMI, and endothelial activations [53]. Studies have shown that in OA, chondrocytes produce a wide range of inflammatory mediators, including adipokines and VCAM-1, leading to cartilage loss. It appears that higher VCAM-1 expression may indicate increased inflammation in the joint tissues [54]. In a different study, Harasymowicz et al. highlight that adiponectin, a pro-inflammatory adipokine in OA, can induce VCAM-1 expression in human OA chondrocytes. In addition, serum levels of soluble VCAM-1 appear to be a strong and independent marker for the likelihood of hip and knee replacements, and they exhibit a positive correlation with hand OA [55]. Our research demonstrated a rise in VCAM-1 levels in the blood following 3 months of vitamin D supplementation. Both AOCs and COCs patients experienced this increase. This is quite probable for our patients who, because of an orthopedic condition restricting mobility along with obesity, experienced significant inflammation in both the AOCs and COCs groups.

The research conducted by Halder et al. found that 1,25(OH)2D3 functions by binding to *VDR*, which leads to a reduction in *PAI-1* protein expression in a human uterine fibroid cell line [56]. Additionally, Barbosa et al. revealed that high *PAI-1* reactivity in fibroblasts decreased in response to 1,25(OH)2D3 [57]. Furthermore, Wu-Wong et al. showed that vitamin D analogs can inhibit PAI-1 in smooth muscle cells (SMCs), but not in endothelial cells (CAECs) [58]. Jorde et al. found significant inverse relationships between 25(OH)D3 and levels of tPA and PAI-1 antigen [59]. Fukomoto et al. reported that 1,25-dihydroxyvitamin D (1,25(OH)2D) lowers PAI-1 production in malignant rat osteoblast cells [60]. In contrast, our results have shown that despite the increase in vitamin D concentration in the examined serum in both study groups, only in the group of patients with AOCs the concentration of PAI-1 in the serum increased. In our group of patients with AOCs, 66% were females. Comparisons between females and males in this group showed that females had higher levels of this marker, even if they were younger than males. In light of the obtained results, it seems that sex may play a significant role in the expression of PAI-1. Studies have shown that PAI-1 is higher in premenopausal women than in men, which is related to genetic and hormonal factors [61]. In contrast, in our study, the comparison of females in the AOCs group and females in the COCs group, who differed statistically significantly in age but showed no difference in PAI-1 levels, correlates with the study cited above, in which premenopausal women and those of menopausal age did not differ statistically in PAI-1 levels [61]. This also aligns with Asselbergs et al.’s research, which indicates that the biology of t-PA and PAI-1 differs between females and males. Age, BMI, and waist-to-hip ratio significantly predicted t-PA and PAI-1 levels in both sexes. The regression relationships connecting these factors to plasma t-PA and PAI-1 varied based on sex [62]. Elevated PAI-1 activity is recognized as a key characteristic of fibrosis. Growing evidence indicates a direct relationship between genetically determined PAI-1 levels and the degree of collagen buildup during injury repair [63].

Topaloglu et al., in their study, examined 75 patients who were divided into three groups based on their 25(OH)D3 levels. They found that TFPI levels were higher in the group with optimal 25(OH)D3 levels (≥20 ng/mL) and showed a strong positive correlation between levels of this vitamin and TFPI [64]. Despite elevated vitamin D levels, no significant difference in TFPI concentration was observed in our patients.

In summary, the subjects included in this study were at risk for thrombosis and inflammation due to obesity. Additionally, orthopedic conditions were another factor that heightened this risk and could influence the various markers studied. However, the study we conducted has several limitations. One significant limitation is the relatively small sample size of subjects. The population was not homogeneous concerning orthopedic conditions, which may have significantly affected the parameters examined. The groups also varied in size regarding sex distribution. It is also essential to consider that age and sex may influence the efficacy of vitamin D. Furthermore, patients may have faced an increased risk for thrombosis and inflammation due to their sedentary lifestyle, which stemmed from limited physical activity associated with orthopedic conditions. The study also did not take into account the lifestyle factors of the subjects, such as type of diet, smoking, and alcohol consumption, which can also significantly affect the markers we studied. Moreover, the outcomes may have been affected by the painkillers and anti-inflammatory medications taken by the patients, as well as different drugs, like hypertension medicines or hormones. Importantly, the influence of other comorbidities on obtained marker concentrations cannot be excluded. It is also essential to consider the phenomenon of vitamin D sequestration in adipose tissue. If this study is carried out, a follow-up study should be conducted to determine the dose and possible further treatment.

## 5. Conclusions

Our research indicated that, despite increased serum vitamin D levels, most patients exhibited elevated markers such as VCAM-1 and PAI-1 following three months of vitamin D supplementation at a dosage of 4000 IU per day. This suggests that, although the anti-inflammatory properties of vitamin D are frequently highlighted in the literature, along with its potential role in modulating epithelial function and influencing coagulation-related markers, it does not appear to provide additional health benefits in obese patients suffering from orthopedic conditions. Additionally, some limiting factors—such as age, sex, comorbidities, medications, diet, physical activity, smoking, or alcohol consumption—should be considered, as these variables may significantly impact our results and need to be included in future research projects.

## Figures and Tables

**Table 1:** Comparison of the mean and standard error of the mean (SEM) of vitamin D and markers in the AOCs group: before [1] and after [2] three-month supplementation.

	AOCs Group		
Protein	Mean ± SEM [1]	Mean ± SEM [2]	*p*-Value
α2AP [ng/mL]	30.45 ± 2.83	29.79 ± 2.38	0.446
TFPI [ng/mL]	159.93 ± 22.46	165.06 ± 22.18	0.184
PAI-1 [ng/mL]	2.51 ± 0.32	2.82 ± 0.38	0.043
VCAM-1 [ng/mL]	13.22 ± 0.52	14.13 ± 0.6	0.007
Vitamin D [ng/mL]	19.44 ± 1.4	33.83 ± 3.12	<0.001

**Table 2:** Comparison of the mean and standard error of the mean (SEM) of vitamin D and markers in the COCs group: before [1] and after [2] three-month supplementation.

	COCs Group		
Protein	Mean ± SEM [1]	Mean ± SEM [2]	*p*-Value
α2AP [ng/mL]	29.09 ± 2.98	30.34 ± 2.81	0.460
TFPI [ng/mL]	149.66 ± 23.95	167.04 ± 21.54	0.112
PAI-1 [ng/mL]	2.35 ± 0.33	2.51 ± 0.34	0.177
VCAM-1 [ng/mL]	12.52 ± 0.38	13.57 ± 0.61	0.031
Vitamin D [ng/mL]	20.39 ± 1.66	35.22 ± 2.46	<0.001

**Table 3:** Markers and vitamin D serum concentrations—AOCs vs. COCs—before supplementation [1].

	AOCs Group	COCs Group	
Protein	Mean ± SEM [1]	Mean ± SEM [1]	*p*-Value
α2AP [ng/mL]	30.45 ± 2.83	29.09 ± 2.98	0.786
TFPI [ng/mL]	159.93 ± 22.46	149.66 ± 23.95	0.731
PAI-1 [ng/mL]	2.51 ± 0.32	2.35 ± 0.33	0.678
VCAM-1 [ng/mL]	13.22 ± 0.52	12.52 ± 0.38	0.849
Vitamin D [ng/mL]	19.44 ± 1.4	20.39 ± 1.66	0.539

**Table 4:** Markers and vitamin D serum concentrations—AOCs vs. COCs—after supplementation [2].

	AOCs Group	COCs Group	
Protein	Mean ± SEM [2]	Mean ± SEM [2]	*p*-Value
α2AP [ng/mL]	29.79 ± 2.38	30.34 ± 2.81	0.986
TFPI [ng/mL]	165.06 ± 22.18	167.04 ± 21.54	0.459
PAI-1 [ng/mL]	2.82 ± 0.38	2.51 ± 0.34	0.678
VCAM-1 [ng/mL]	14.13 ± 0.6	13.57 ± 0.61	0.551
Vitamin D [ng/mL]	33.83 ± 3.12	35.22 ± 2.46	0.842

**Table 5:** Serum markers and vitamin D concentrations before supplementation [1] in the group with AOCs, categorized by sex.

	Females (*N* = 12)	Males (*N* = 6)	
Protein	Mean ± SEM [1]	Mean ± SEM [1]	*p*-Value
α2AP [ng/mL]	33.49 ± 3.80	24.38 ± 2.66	0.13
TFPI [ng/mL]	189.89 ± 30.14	100.01 ± 8.4	0.06
PAI-1 [ng/mL]	2.89 ± 0.43	1.75 ± 0.12	0.09
VCAM-1 [ng/mL]	13.74 ± 0.64	12.16 ± 0.79	0.16
Vitamin D [ng/mL]	19.99 ± 1.75	18.33 ± 2.49	0.59

**Table 6:** Serum markers and vitamin D concentrations after supplementation [2] in the group with AOCs, categorized by sex.

	Females (*N* = 12)	Males (*N* = 6)	
Protein	Mean ± SEM [2]	Mean ± SEM [2]	*p*-Value
α2AP [ng/mL]	32.16 ± 2.97	25.05 ± 3.47	0.16
TFPI [ng/mL]	194.43 ± 29.84	106.31 ± 8.21	0.06
PAI-1 [ng/mL]	3.37 ± 0.5	1.7 ± 0.13	0.03
VCAM-1 [ng/mL]	14.79 ± 0.75	12.79 ± 0.83	0.12
Vitamin D [ng/mL]	33.82 ± 3.87	33.85 ± 5.79	0.99

**Table 7:** Comparison of markers and vitamin D concentrations between females in the AOCs and COCs groups—before supplementation [1].

	Females (*N* = 12) AOCs Group	Females (*N* = 14) COCs Group	
Protein	Mean ± SEM [1]	Mean ± SEM [1]	*p*-Value
α2AP [ng/mL]	33.49 ± 3.8	29.44 ± 3.18	0.52
TFPI [ng/mL]	189.89 ± 30.16	151.83 ± 25.62	0.52
PAI-1 [ng/mL]	2.89 ± 0.43	2.36 ± 0.36	0.34
VCAM-1 [ng/mL]	13. 74 ± 0.64	12.55 ± 0.4	0.12
Vitamin D [ng/mL]	19.99 ± 1.75	19.89 ± 1.7	0.97

**Table 8:** Comparison of markers and vitamin D concentrations between females in the AOCs and COCs groups—after supplementation [2].

	Females (*N* = 12) AOCs Group	Females (*N* = 14) COCs Group	
Protein	Mean ± SEM [2]	Mean ± SEM [2]	*p*-Value
α2AP [ng/mL]	32.16 ± 2.97	30.21 ± 3.01	0.59
TFPI [ng/mL]	194.43 ± 29.84	168.29 ± 23.1	0.78
PAI-1 [ng/mL]	3.37 ± 0.5	2.53 ± 0.36	0.25
VCAM-1 [ng/mL]	14.79 ± 0.75	13.56 ± 0.66	0.29
Vitamin D [ng/mL]	33.82 ± 3.87	35.75 ± 2.58	0.67

## Data Availability

The original contributions presented in this study are included in the article. Further inquiries can be directed to the corresponding author.
